# Long-term results after surgical basal cell carcinoma excision in the eyelid region: revisited

**DOI:** 10.1007/s00508-024-02333-5

**Published:** 2024-02-13

**Authors:** Reinhard Told, Adrian Reumueller, Judith Kreminger, Birgit Lackner, Andreas Kuchar, Ursula Schmidt-Erfurth, Roman Dunavoelgyi

**Affiliations:** Department of Ophthalmology and Optometry, Medical University of Vienna, Spitalgasse 23, 1090 Vienna, Austria

**Keywords:** Basal cell carcinoma, Eyelid, Surgical excision, Recurrence rate, Periocular

## Abstract

The aim of the study was to readdress basal cell carcinoma (BCC) in the periocular region to prove the efficacy of histologically controlled surgical treatment and to identify high-risk characteristics.

Retrospective analysis of 451 microscopically controlled BCC excisions in the periocular region. Tumor location, tumor size, AJCC 7 classification, and histological results were recorded. The same procedure was followed for recurrences.

A recurrence rate of 5.0% was observed after the first microscopically controlled excision. Recurrent BCCs show a shift from nodular to sclerosing BCC as the primary histological type as well as a change in primary location from lower eyelid to medial canthus. The frequency of BCC with deep extension increased from 7.3% to 24.7%, and 57.1% after the second and third operations, respectively. The recurrence rate increased to 9.5% and 42.9%, after the second and third operations, respectively.

In conclusion, we are facing the same challenges in surgical BCC treatment as 30 years ago. The distribution of periocular BCC location, histologic subtype and recurrence rates mirror the literature und the general consensus. The recurrence rate increases with every operation needed. Sclerosing BCCs with deep extension at the medial canthus bear the greatest risk for recurrence. In such cases, centers of expertise should be consulted and additional treatment options should be considered.

## Background

Data from the American Cancer Society show that basal cell carcinoma (BCC) of the skin accounts for half of all cancers diagnosed in the USA [1]. Approximately 5–10% of all skin cancers occur in the eyelids [2]. Within this anatomical region, BCC accounts for 80–95% of all eyelid cancers [3], with an age-standardized incidence of 4.5 [4] and a prevalence of 87.5 per 100,000 inhabitants [5]. Regional differences in numbers are well documented, with incidences of 1772 per 100,000 peaking in Queensland Australia [6]. Exposure to UV radiation, fair skin types, and age [7] are considered important risk factors. Furthermore, sex, race, ethnicity, and smoking status were positively associated with BCC based on the analysis of 82,136 patients with eyelid cancers [5].

The neoplastic transformation of the basal cells of the epidermis marks the origin of BCC. The transformed cells proliferate and invade the dermis and adjacent tissues, forming nodules, invasive strands or tissue-mimicking adnexal structures [2]. The BCCs rarely metastasize, have been reported in up to 1% of cases and can occur through hematogenous and lymphatic pathways [8]. A meta-analysis has rated evidence levels for BCC treatment options. Mohs’ micrographic surgery (MMS) and excision with frozen section control have a strong (level 1) rating followed by substantial ratings (level 2) for photodynamic therapy, carbon dioxide laser treatment, electron beam radiotherapy, chemotherapy, and cryotherapy in localized BCCs [2, 9]. The 5‑year cure rate after MMS is reported to be 99% after primary BCC excision and 94% for recurrent BCC in the periocular region [6].

We have previously shown that BCC with deep extension located at the medial canthus of the eye bears the greatest risk of recurrence. This finding was based on the evaluation of 382 patients with BCC between 1980 and 1993 [10]. As BCC is still the most common skin cancer in the periocular region, 30 years later we have reassessed the long-term results after surgical BCC excision.

## Methods

The Ethics Committee of the Medical University of Vienna, Vienna, Austria approved the protocol of the present study (EK:1997/2019). As this study is of a retrospective nature, the Ethics Committee of the Medical University of Vienna waived the need for informed consent. The study was conducted in adherence to the Declaration of Helsinki including current revisions and the good clinical practice (GCP) guidelines.

Patient records were reviewed for the presence of BCC and surgical excision with frozen section control of the wound margins in the periocular region, conducted between 1 January 2009 and 31 December 2019. Periocular was defined as the area involving the medial and lateral canthus as well as the upper and lower eyelids. The BCCs outside this region were excluded from this analysis, as well as those with incomplete records. For comparative reasons, we adopted the previously introduced groups [10] based on tumor size, which are: 1–4 mm, 5–9 mm, 10–14 mm, 15–19 mm, and 20 mm or larger. Infiltration of the orbicularis oculi muscle was recorded separately as BCC with depth extension. Additionally, we have grouped BCCs according to the American Joint Committee on Cancer (AJCC 7) carcinoma of the eyelid TNM (tumor-nodes-metastasis) definitions [11]: 0–5 mm (T1), > 5–10 mm (T2a), > 10–20 mm (T2b), > 20 mm (T3a).

The BCC subtypes were classified based on histopathological growth pattern and histological differentiation [12], which are characterized by the presence of cellular and stromal elements as well as by the level of cellular differentiation. The BCCs were grouped as nodular, sclerosing (morphea-like), and superficial micronodular (previously also known as superficial multicentric) BCCs, in order to address the evolution in BCC classification [13], but also to allow comparison to previous results [10].

### Surgical procedure

Operations were performed with the patient under local anesthesia. If local anesthesia was not tolerated by the patient or was insufficient due to the extent of the tumor, general anesthesia was used. Magnetic resonance imaging was used in cases of widespread or infiltrating tumors for planning the operation.

In short, we adhered to the principles of an approach synonymously known as surgical excision with microscopic tissue margin control, chemosurgery fresh-tissue technique, surgical excision with microscopic control or Mohs’ surgery—fresh tissue technique [14–16] but not performed by trained Mohs’ surgeons. First, the visible tumor is excised aiming at a safety margin of resection of 4–5 mm [17], smaller margins are taken (2–3 mm) in areas where reconstructive options are limited. Second, thin layers of tissue in a saucer-like shape [16] circumferential to the defect are excised, a drawing of the tumor is sketched, labeling and color mapping are added for detailed orientation. Frozen sections are analyzed [16] by histopathology specialists and results are reported back to the surgeon based on the map until total removal of the tumor is achieved.

Primary wound closure is achieved by same day direct skin closure, including undermining wound margins if necessary, local transposition flaps, or free skin transplants from the upper eyelid, the preauricular or postauricular region with or without fibrin glue tissue fixation.

Follow-up examinations were performed routinely 1 week, 1, 3, 6 and 12 months after surgical excision, followed by annual check-ups for up to 3 years or longer if necessary.

### Statistics

Data were reported descriptively. The t‑test was used to compare means between groups. Bivariate correlation was used for ordinal parameters. SPSS (Version 26.0, IBM, Armonk, NY, USA) was used.

## Results

We identified 451 patients with histologically confirmed BCC between 2009 and 2019. Of the patients 398 were scheduled for primary tumor excision and 53 patients were referred to our department for BCC recurrence after undergoing primary BCC excision elsewhere.

Mean patient age was 72.5 ± 12.8 years, 62.8% (283) were female and 37.2% (168) were male. At the time of the operation the mean female patient age was 73.1 ± 13.0 years and the mean male patient age was 71.5 ± 12.4 years (*p* = 0.92). In 52.3% (208) of patients the right eye was affected, in 47.7% (190) the left eye. The mean time of follow-up was 3 ± 2 years (range 1–10 years).

### Primary operation

**Fig. 1:**
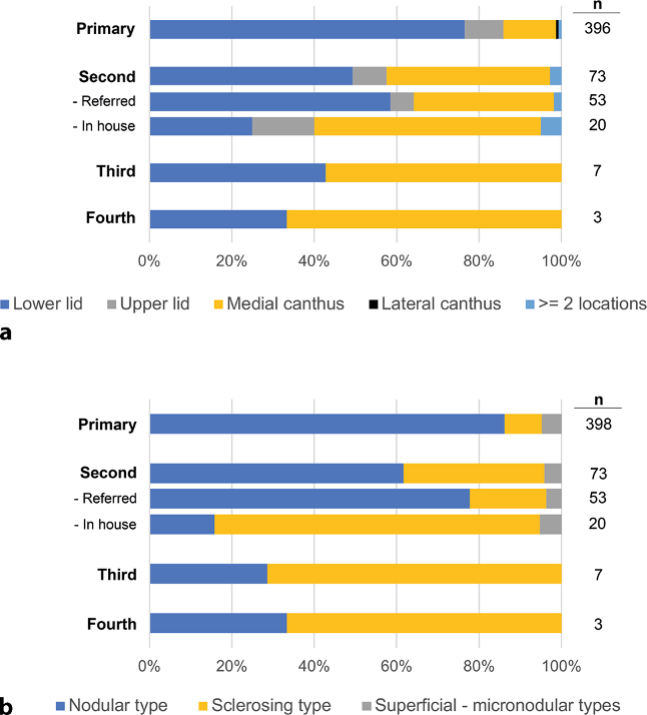
Basal cell carcinoma (BCC).** a** Periorbital BCC location distribution (%) displayed for primary and subsequent operations. **b** Frequencies of histological BCC types displayed for primary and subsequent operations. *n* number of subjects

The BCC locations were distributed as follows (see Fig. 1a); 76.1% (303) on the lower eyelids, 12.8% (51) on the medial canthus, 9.3% (37) on the upper eyelids, 0.5% (2) lateral canthus and 0.8% (3) had 2 locations affected by BCC, all of which were located at the lower eyelids and medial canthus. In two patients the exact location, apart from the eye, was not documented in the record.

Lesion size categorized as previously described [10] showed that 13.3% (53) were between 1–4 mm, 37.4% (149) were between 5–9 mm, 25.9% (103) were between 10–14 mm, 9.3% (37) were between 15–19 mm, 6.8% (27) were over 20 mm in greatest linear diameter and 7.3% (29) showed depth extension.

Grouping BCC based on the AJCC 7 TNM classification revealed that 20.9% (83) were stage T1, 45.5% (181) stage T2a, 27.6% (110) stage T2b, and 6.0% (24) reached stage T3a.

Histological examination showed that 86.2% (343) of the primary tumors were nodular types, 9.0% (36) were sclerosing types and 4.8% (19) were superficial micronodular types.

Bivariate correlation of AJCC 7 T staging and histology showed a very weak but positive correlation (Spearman: 0.12, *n* = 398).

In over 80% of surgeries primary wound closure was achieved either by direct wound closure (45.2%, 180) or by local transposition flaps (41.5%, 165). In 13.3% (53) of the patients free autologous skin transplants, both with and without fibrin sealing were used for wound closure.

Complications were recorded in 9.3% (37) of patients, which were wound dehiscence (6.4%) and ectropion (2.9%) and 4.5% (18) of patients underwent revision surgery due to wound dehiscence.

### Second operation

In total 73 (16.2%) of the 451 patients underwent surgery due to BCC recurrence. Of the 398 patients with a primary operation performed at our department, the recurrence rate was 5.0% (*n* = 20).

The lesion size shows that 8.2% (6) were between 1–4 mm, 42.5% (31) were between 5–9 mm, 21.9% (16) were between 10–14 mm, 5.5% (4) between 15–19 mm, 2.7% (2) were over 20 mm in greatest linear diameter; 19.2% (14) showed depth extension.

Grouping recurring BCCs based on the AJCC 7 TNM classification showed that 24.7% (18) were stage T1, 47.9% (35) stage T2a, 26.0% (19) stage T2b, and 1.4% (1) reached stage T3a. Bivariate correlation of AJCC 7 T staging and histology showed a very weak correlation (Spearman: 0.03, *n* = 73).

Histological analyses of these first recurrences show that 61.6% (45) were nodular types, 34.3% (25) were sclerosing BCC, and 4.1% (3) were superficial-micronodular types.

### Third operation

In the group of 73 secondary operations the recurrence rate was 9.5% (7). In four patients, the tumor was located at the medial canthus and in three patients at the lower eyelid. The tumor was classified as sclerosing in five patients and as nodular in two patients (see Fig. 1a, b for percent distribution) and four were classified as having depth extension.

### Fourth operation

In the group of 7 patients undergoing a third operation, three (42.9%) had to undergo a fourth operation; two were located at the medial canthus and one at the lower eyelid; two were classified as sclerosing type BCC and one as nodular type BCC. See Fig. 1a, b for percent distribution. One patient showed depth extension.

One patient with sclerosing type BCC located at the medial canthus finally underwent vismodegib treatment approximately 7 years after the first contact, as no functionally acceptable result could be achieved.

## Discussion

In this study we have readdressed the long-term results after surgical BCC excision in the periocular region. Today surgical BCC excision and in particular MMS is considered the gold standard in BCC treatment, despite a plethora of alternative treatments available, such as cryotherapy, chemotherapy and radiotherapy [2, 9]. Considering an increasing life-expectancy [18] over the last decades associated with increasing numbers of eyelid BCC with increased age, this research question is still topical.

Patient and BCC characteristics are in accordance with our previous report [10]. The majority of patients were between 60 and 80 years old, with a mean age of 72.5 ± 12.8 years. The two most common locations were the lower eyelid and the medial canthus, which is also in line with the literature [19–26]. Greatest linear diameter of the BCC was 5–9 mm in 37.4% or as defined by the AJCC 7 classification 45.5% reached stage T2a. Greatest linear diameter is also in accordance with our previous report where the majority (40.9%) was 5–9 mm in size at initial presentation [10].

The number of nodular BCCs increased from 73% to 86%, whereas sclerosing BCCs decreased from 14% to 9%, yet the frequency distribution did not change compared to our previous report [10]. Hence, a 60–80 year old patient with 5–9 mm lesion at the lower eyelid or medial canthus, with a nodular or sclerosing appearance should lead to a high suspicion for BCC.

A recurrence rate of 5.0% was observed after a mean follow-up of 3 ± 2 years for the 398 patients whose primary BCC resection was performed at our department. The recurrence rate increases with each additional operation needed. In this study recurrence rates were 9.5% and 42.9% for the third and fourth operation needed, respectively. It is further evident that with every additional operation needed, the distribution regarding BCC location changes from lower eyelid to medial canthus (see Fig. 1a) as the most common site of recurrence. Also, histological types change from nodular to sclerosing BCC as the most common histological BCC type. The relatively high number of nodular BCCs at the timepoint of the first revision surgery may be explained by the number of referrals for excision surgery (see Fig. 1b: referred). Additionally, the number of BCCs with depth extensions increased from 7.3% at primary excision surgery to 19.2%, and 57.1% at the second and third operation, respectively. Finally, from the three patients undergoing a fourth operation, one (33.3%) showed depth extension. These findings are in line with previous reports regarding histological types and location, as well as recurrence rates ranging between 1.5% and 9.2% in the literature [19–26].

Caution is advised when comparing recurrence numbers as surgical techniques and follow-up periods differ vastly. As most BCCs recur within 3 years after treatment and up to one third after 5 years [25, 27], sufficient follow-up time is essential in clinical studies. Filtering studies for surgical approach and follow-up time shows that BCC recurrence rate after MMS is even lower, between 1.5% and 5%. [10, 23, 25, 26, 28–30]. Studies with longer follow-up periods (4 and 5 years, respectively) reported marginally higher numbers between 2% and 5% [30, 31] highlighting that 3 years of follow-up to be a valuable follow-up period, which also applies to this study.

The change in primary location of occurrence, histologic type and increase in numbers with depth extension with each consecutive operation performed, may be attributed to the anatomical structures comprising the canalicular system, medial canthal tendons as well as the insertions of the orbital septum, which may lead to cautious BCC and circumferential tissue excision. This has been confirmed in a multicenter study, where oculoplastic surgeons were more likely than plastic surgeons or dermatologists to incompletely excise BCCs, which was attributed to the more difficult case mix regarding location [32]. There is still debate on the ideal size of safety margins. It has been shown that an 8‑mm margin would completely remove 95% of high-risk BCCs [17], which is not possible in most periocular cases. This dilemma is further supported by the finding that in patients with BCCs the mean margin inside the H‑zone was significantly smaller than that outside the H‑zone [17]. Using different sets of instruments for the excision of the tumor, layered tissue segments and reconstruction regarding recurrence have been discussed previously [10] and should be considered a standard procedure. These principles are followed at our institution and may therefore be neglected in this discussion.

The AJCC 7 T stages and histological BCC types only show a very weak to no correlation, both at primary and secondary excision, and do not allow conclusions on suspected type based on size. We used the AJCC 7 T classification as it was available between 2009 and 2019. The current AJCC 8 was established in 2016 [33].

Limitations to be considered are the retrospective nature of this study, the use of AJCC 7 and the limited comparability to other studies due to various follow-up periods, as discussed above. Also, we cannot report the number of resections necessary to get negative margins retrospectively, as patient records are incomplete regarding this specific question.

Previously we have concluded that “In our opinion microscopic control of wound margins ought to become standard practice in the surgical treatment of BCC.” [10], today we can state that with the widespread use of MMS it has become a standard in BCC treatment, especially when it comes to retreatment, with abundant literature supporting its use [2].

In conclusion, we are facing the same challenges in surgical BCC treatment as 30 years ago. The distribution of periocular BCCs and histological subtypes to date mirror the literature und the general consensus. BCC excision at the medial canthus may be challenging, particularly in high-risk cases, such as recurrent sclerosing BCC with deep extensions. In such cases, centers of expertise should be consulted and additional treatment options such as radiation therapy or systemic therapy may be considered.

## Data Availability

The datasets generated and/or analyzed during the current study are not publicly available as the ethics vote does not include sharing data publicly; however, data are available from the corresponding author on reasonable request.

## Abbreviations

AJCC7: The American Joint Committee on Cancer: the 7th edition of the AJCC cancer staging manual: tumor
BCC: Basal cell carcinoma
GCP: Good clinical practice
MMS: Mohs’ micrographic surgery
TNM: Tumor-nodes-metastasis
